# Parthanatos and apoptosis: unraveling their roles in cancer cell death and therapy resistance

**DOI:** 10.17179/excli2025-8251

**Published:** 2025-03-04

**Authors:** Gaurav Gupta, Muhammad Afzal, Ehssan Moglad, Ahsas Goyal, Waleed Hassan Almalki, Kavita Goyal, Mohit Rana, Haider Ali, Arcot Rekha1, Imran Kazmi, Sami I. Alzarea, Sachin Kumar Singh

**Affiliations:** 1 Centre for Research Impact & Outcome, Chitkara College of Pharmacy, Chitkara University, Rajpura, Punjab 140401, India; 2 Centre of Medical and Bio-allied Health Sciences Research, Ajman University, Ajman, United Arab Emirates; 3 Department of Pharmaceutical Sciences, Pharmacy Program, Batterjee Medical College, P.O. Box 6231, Jeddah 21442, Saudi Arabia; 4 Department of Pharmaceutics, College of Pharmacy, Prince Sattam bin Abdulaziz University, Alkharj 11942, Saudi Arabia; 5 Institute of Pharmaceutical Research, GLA University, Mathura, UP, India; 6 Department of Pharmacology, College of Pharmacy, Umm Al-Qura University, Makkah, Saudi Arabia; 7 Department of Biotechnology, Graphic Era (Deemed to be University), Clement Town, 248002, Dehradun, India; 8 Uttaranchal Institute of Pharmaceutical Sciences, Uttaranchal University, Dehradun, India; 9 Centre for Global Health Research, Saveetha Medical College, Saveetha Institute of Medical and Technical Sciences, Saveetha University, Chennai, India; 10 Dr. D.Y. Patil Medical College, Hospital and Research Centre, Pimpri, Pune, India; 11 Department of Biochemistry, Faculty of Science, King Abdulaziz University, Jeddah 21589, Saudi Arabia; 12 Department of Pharmacology, College of Pharmacy, Jouf University, Sakaka 72341, Al-Jouf, Saudi Arabia; 13 School of Pharmaceutical Sciences, Lovely Professional University, Phagwara, Punjab 144411, India; 14 Sunway Biofunctional Molecules Discovery Centre (SBMDC), School of Medical and Life Sciences, Sunway University, Sunway, Malaysia

**Keywords:** parthanatos, apoptosis, cancer, cell death mechanisms, PARP1, caspases, therapy resistance

## Abstract

Cell death is a fundamental process that needs to be maintained to balance cellular functions and prevent disease. There are several cell death pathways; however, apoptosis and parthanatos are the most prominent and have important roles in cancer biology. As an extremely well-regulated process, apoptosis removes damaged or abnormal cells via caspase activation and mitochondrial involvement. Unlike in the healthy cells, the loss of ability to induce apoptosis in cancer permits tumor cells to survive and multiply out of control and contribute to tumor progression and therapy resistance. On the contrary, parthanatos is a caspase-independent metabolic collapse driven by poly (ADP-ribose) polymerase 1 (PARP1) overactivation, translocation of apoptosis-inducing factor (AIF), and complete DNA damage. Several cancer models are involved with parthanatos. Deoxypodophyllotoxin (DPT) induces parthanatos in glioma cells by excessive ROS generation, PARP1 upregulation, and AIF nuclear translocation. Like in acute myeloid leukemia (AML), the cannabinoid derivative WIN-55 triggers parthanatos, and the effects can be reversed by PARP inhibitors such as olaparib. Developing cancer treatment strategies involving advanced cancer treatment strategies relies on the interplay between apoptosis and parthanatos. However, such apoptosis-based cancer therapies tend to develop resistance, so there is an urgent need to look into alternative pathways like parthanatos, which may not always trigger apoptosis. In overcoming apoptosis resistance, there is evidence that combining apoptosis-inducing agents, such as BH3 mimetics, with PARP inhibitors synergistically enhances cell death. Oxidative stress modulators have been found to promote the execution of parthanatic and apoptotic pathways and allow treatment. In this review, apoptosis and parthanatos are thoroughly compared at the molecular level, and their roles in cancer pathogenesis as related to cancer therapeutic potential are discussed. We incorporate recent findings to demonstrate that not only can parthanatos be used to manage therapy resistance and enhance cancer treatment via the combination of parthanatos and apoptosis but also that immunity and bone deposition can feasibly be employed against long-circulating cancer stem cells to treat diverse forms of metastatic cancers.

## Introduction

Cellular homeostasis is maintained, damaged or aberrant cells are eliminated, and proper development is assured through the important biological process of programmed cell death (Qian et al., 2024[120]). Apoptosis was widely investigated in various forms of programmed cell death due to its important role in eliminating damaged cells irreparably and ensuring that tissue and organismal health are protected (Shkarina and Broz, 2024[129]). However, cell death dysregulation is a hallmark of cancer, a hall that allows the advancement of tumors, exposure to chemotherapies, and tumors' avoidance of immune attacks (Tufail et al., 2024[141]). Typically, the intrinsic balance between pro-survival and pro-death signals is often perturbed in cancer, allowing it to evade immune surveillance and respond to therapies that involve pro-apoptotic activation (Plati et al., 2011[118]). However, the unity in diversity in the controls of apoptosis and alternative cell death pathways, namely parthanatos, is extremely important to understand in order to develop effective and targeted cancer treatments (Zhou et al., 2021[181]). Apoptosis is defined by certain morphological indicators, including DNA breakage, chromatin condensation, and the production of apoptotic bodies; it is thought to be mediated exclusively by a caspase-dependent process (Kroemer et al., 2009[75]). The apoptotic process is tightly controlled by both extrinsic (death receptor) and intrinsic (mitochondrial) signaling cascades, which are carried out by caspases, cytochrome c, and Bcl 2 family members (Xu and Shi, 2007[163]). Then, parthanatos, a relatively newly identified form of programmed necrosis, is a caspase-independent process activated by the overactivation of PARP1 (Fatokun et al., 2014[42]). When severe DNA damage triggers PARP-1 hyperactivity, the intrinsic mechanisms cause PAR polymer buildup, mitochondrial malfunction, PARP-1 nuclear translocation, large-scale DNA fragmentation, and cell death (Wang et al., 2009[151]). However, parthanatos is different because it is not immunologically silent, as in apoptosis, and can contribute to inflammatory signaling that, in turn, modifies the tumor microenvironment and therapeutic consequences (Gielecińska et al., 2023[50]).

The therapeutic relevance of the distinction between apoptosis and parthanatos is based on the fact that they have complementary functions in cancer treatment (Su et al., 2016[135]). Many tumors avoid apoptosis due to mutations in tumor suppressor genes like TP53 and overexpression of anti-apoptotic proteins like Bcl2 and IAPs (Crowe and Sinha, 2006[32]). The resistance conferred by these alterations is due to conventional therapies that depend upon caspase activation (Boice and Bouchier-Hayes, 2020[13]). BH3 mimetics, for example, venetoclax, designed to restore apoptotic sensitivity by inhibiting Bcl-2, may be efficacious only in apoptosis-resistant cancers (Montero and Haq, 2022[108]). Therefore, targeting parthanatos is a promising alternative in such cases (Zhang et al., 2024[172]). Deoxypodophyllotoxin (DPT) and WIN-55 can also effectively trigger parthanatos in gliomas and AML, even in tumors unresponsive to apoptotic-based treatment (Ma et al., 2016[98]). Parthanatos is particularly relevant in both situations in which parasites are known to have roles, such as cancers being treated with DNA-damaging agents like platinum-based drugs and radiation (Huang et al., 2022[59]). A shortage of ATP and NAD+, mitochondrial malfunction, and, eventually, cell death are caused by PARP-1 hyperactivation in response to severe DNA damage (Murata et al., 2019[110]). To inhibit BRCA-mutated cancers, PARP inhibitors were first afforded as synthetic lethality inducers (Lord and Ashworth, 2017[93]). These inhibitors have been found to selectively induce parthanatos in tumor cells with faulty DNA repair mechanisms (Xu et al., 2023[166] ). This has created new combinatorial opportunities for killing resistant cells in cancer by combining apoptosis and parthanatos (Zhou et al., 2021[181]). 

In contrast, blending PARP inhibitors with BH3 mimetics or immune checkpoint inhibitors has synergistic effects by activating multiple cell death pathways, improving treatment efficacy, and reducing resistance (Wu et al., 2021[160]). In addition to their therapeutic potential, they have diagnostic and prognostic importance concerning such distinction between apoptosis and parthanatos (Maru et al., 2023[100]). Because apoptotic and parthanatic pathways share distinct biochemical and morphological markers, these can be used as biomarkers of treatment response (Mustafa et al., 2024[111]). Furthermore, the relationship between metabolic stress, mitochondrial malfunction, oxidative stress, and the reciprocal activities of parthanatos and apoptosis may result in new treatment approaches that take advantage of this interaction in a tumor-specific manner (Chen et al., 2011[25]). There are two distinct, though therapeutically complimentary, ways of programmed cell death: apoptosis and parthanatos (Andrabi et al., 2008[5]). Even though cancer therapy has long focused on apoptosis, a topic of increasing awareness is that parthanatos has brought new avenues for circumventing resistance to therapy, genomic instability, and metabolic adaptations of tumors (Tong et al., 2022[139]). Cancer can be targeted through these pathways to develop more effective, personalized, and durable therapeutic strategies by distinguishing them and probing how they interact. This review synthesizes molecular mechanisms, pathological roles, and therapeutic significance of apoptosis and parthanatos, two prominent pathways regulating cellular death. It describes their impact on understanding and treating cancer biology and treatment.

## Apoptosis: A Classical Programmed Cell Death

### Mechanistic insights into apoptosis

Apoptosis is a basic process that preserves cellular homeostasis by getting rid of damaged or unnecessary cells, which can also be potentially risky for the organism (Ameisen, 2002[3]). Unlike necrosis, a non-regulated and inflammatory means of cell destruction, cell death by apoptosis is a tightly controlled and non-inflammatory process whereby cell debris contained within apoptotic bodies is removed by phagocytes (Xu et al., 2019[165]). Without this tightly controlled mechanism, normal development, immune regulation, and cancer suppression would not have occurred (Vinay et al., 2015[142]). The intrinsic and extrinsic routes are the two main ways that apoptosis is carried out, and they both lead to a caspase cascade of proteolytic enzymes (Jan and Chaudhry, 2019[62]).

The primary triggering signals of the intracellular route are intercellular stress signals, including oxidative stress, DNA damage, and nutritional scarcity (Su et al., 2024[134]). They trigger the oligomerization and leakage of the outer membrane of the mitochondria by proapoptotic members of the Bcl-2 protein family, such as Bak and Bax (Kale et al., 2018[65]). Cytochrome c is released into the cytosol due to this MOMP, where it binds Apaf-1 and procaspase-9 to create the apoptosome (Garrido et al., 2006[49]). The apoptosome's ability to activate caspase-9 opens the door for more caspase cleavage and the stimulation of executioner caspases, such as caspase-3 and caspase-7 (Brentnall et al., 2013[15]). DNA breakage, chromatin condensation, cytoskeletal breakdown, and the creation of apoptotic bodies are the outcomes of apoptotic caspases' execution of a crucial cellular component degradation (Povea-Cabello et al., 2017[119]). Specifically, anti-apoptotic proteins like Bcl-2 and Bcl-xL inhibit apoptosis in healthy cells by opposing Bax and Bak, preserving the integrity of mitochondria (Luna-Vargas and Chipuk, 2016[96]). Both anti-apoptotic proteins are over-expressed in cancer to drive tumor cells to avoid apoptosis.

**Figure 1:**
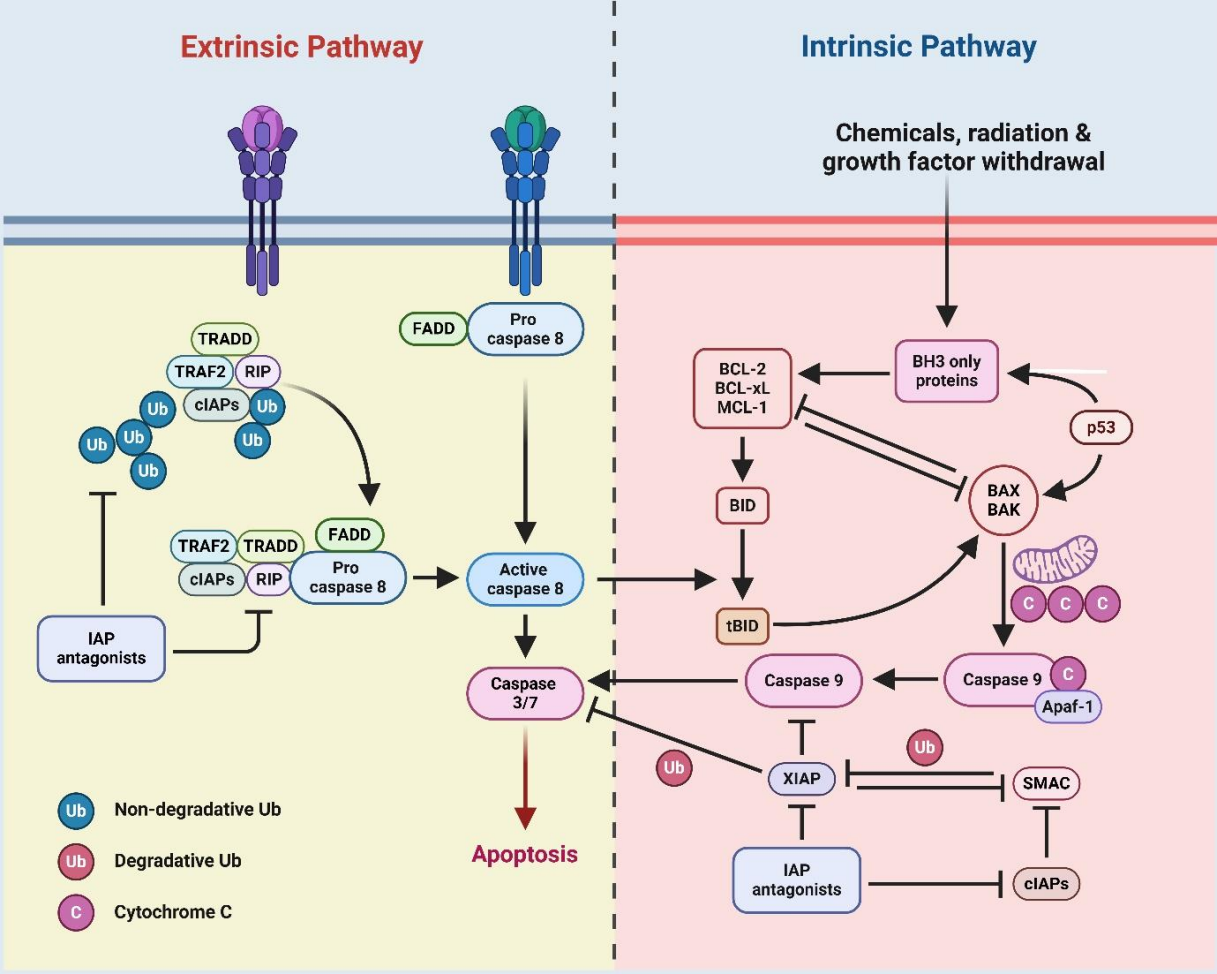
This figure illustrates the extrinsic and intrinsic pathways of apoptosis. The extrinsic pathway is triggered by death receptor activation, leading to the recruitment of adaptor proteins (TRADD, TRAF2, RIP) and activation of caspase-8, which subsequently activates caspases-3/7 to induce apoptosis. The intrinsic pathway is initiated by cellular stress factors such as chemicals, radiation, and growth factor withdrawal, leading to mitochondrial outer membrane permeabilization via BAX/BAK activation. This releases cytochrome C, which activates caspase-9 through Apaf-1, further activating caspases-3/7. Both pathways are regulated by inhibitors of apoptosis proteins (IAPs) and antagonists like SMAC.

On the other hand, the extrinsic pathway is triggered by the extracellular death signal via activation of the death receptor, for instance, Fas (CD95) or tumor necrosis factor receptor (TNFR) upon ligand binding (Guicciardi and Gores, 2009[52]). The DISC is formed due to the binding of adaptor proteins such as procaspase-8 and the death domain-containing protein FADD (Siegmund et al., 2001[131]). After activation, caspase 8 either cleaves and activates executioner caspases directly or cleaves a proapoptotic Bcl2 family member, such as Bid, which releases Mito cytochrome c, activating the intrinsic pathway (Zhong et al., 2020[178]). Effective cell death guarantees that the apoptotic signal is amplified by further cross-talk between the intrinsic and extrinsic pathways (Roy and Nicholson, 2000[125]). In addition, some cancer cells can use mutations in death receptor signaling or downregulation of Fas and TNFR expression to evade apoptosis, thereby evading the immune system and becoming resistant to therapy (Leonard and Johnson, 2018[82]). Though caspase-dependent apoptosis is typically considered, caspase independence mechanisms exist, especially when caspase activation is impaired (McIlwain et al., 2013[102]). Instead, induction of massive DNA fragmentation and chromatin condensation occurs independently of caspases through mitochondrial factors such as apoptosis triggering factor and endonuclease G, which go into the nucleus (Bajt et al., 2006[9]). Under stress conditions, such non-canonical apoptotic pathways ensure that cells die in an active, programmed fashion even if primary apoptotic mechanisms are blocked to maintain cellular integrity (Mustafa et al., 2024[111]) (Figure 1(Fig. 1)).

### Dysregulation of apoptosis in cancer

Apoptosis is a fundamental aspect that helps keep the tissue balanced and prevents the cells with oncogenic potential from surviving (Wong, 2011[155]). However, cancer is a disease of dysregulated apoptosis, a hallmark of cancer allowing tumor cells to avoid programmed cell death, grow in an uncontrolled manner, and limit their susceptibility to therapy (Pistritto et al., 2016[117]). Cancer cells can escape apoptosis in many different ways. However, genetic changes in tumor suppressor genes, including TP53, hamper this, which stops the intrinsic pathway from activating and proapoptotic proteins like Bax and Bak from activating (Dandoti, 2021[35]). Bcl-2, Bcl-xL and Mcl-1 are anti-apoptotic proteins overexpressed in many tumors and interfere with the apoptotic signal while maintaining mitochondrial integrity (Carrington et al., 2017[18]). IAPs also contribute to cancer cell survival by inhibiting caspase activation, and some tumor cells downregulate death receptors or deviant the shape of DISC, impairing the extrinsic apoptotic pathway (Derakhshan et al., 2017[38]).

In addition, these alterations enhance both tumorigeneses and therapy resistance (Dzobo et al., 2023[41]). Tumors that have become resistant to apoptotic signaling often exhibit poor responses to treatment because many chemotherapeutic agents and treatments based on radiation therapy are acting by inducing apoptosis (Wu et al., 2023[159]). For example, most TP53 mutated cancers resist DNA-damaging agents due to p53's lack of function to initiate the intrinsic apoptotic cascade (Wang et al., 2024[145]). Like those tumors, tumors that overexpress Bcl-2 or IAPs escape chemotherapy-induced apoptosis by perturbing the activation of caspases and reducing the era of chemotherapy (Buchholz et al., 2003[16]). 

To counteract these mechanisms, efforts toward developing new cancer therapies are underway to restore apoptotic signaling (Tian et al., 2024[138]). One method that has been proven to be effective is using small molecule inhibitors known as BH3 mimetics to treat anti-apoptotic proteins. These inhibitors neutralize Bcl 2 family members, allowing pro-apoptotic factors to initiate cell death (Delbridge and Strasser, 2015[37]). Since then, it has shown great efficacy in treating hematological malignancies like, for example, CLL and AML (Lasica and Anderson, 2021[79]). In addition, the intrinsic apoptotic pathway is activated through death receptor agonists (including TRAIL receptor agonists) and death receptor agonists that selectively induce tumor cell death (Yuan et al., 2018[171]). Another emerging strategy is using SMAC mimetics, which antagonize IAPs to enhance caspase activation further and increase apoptotic sensitivity in resistant tumors (Wu et al., 2007[156]).

The combination therapies utilizing these agents alone or combined with conventional treatments like chemotherapy, immune checkpoint inhibitors, or targeted therapies are promising approaches to overcome resistance and improve treatment efficacy (Patel and Minn, 2018[115]). It is well known that BH3 mimetics and PARP inhibitors alone effectively kill apoptosis-resistant tumors via both apoptotic and parthanatic pathways (Townsend et al., 2021[140]). Oxidative stress modulators have been found to sensitize tumors to apoptosis-inducing agents, like those that increase mitochondrial dysfunction (Kuo et al., 2022[77]). The development of effective treatments depends on understanding apoptotic mechanisms, and these multi-target approaches highlight the reality that many of the efforts to eliminate cancer cells are focused on targeting the apoptosis-related cell death cascade (Neophytou et al., 2021[112]).

## Parthanatos: A Poly(ADP-Ribose)-Dependent Cell Death

### Molecular signature of parthanatos

The paradigm of overactivation of the PARP-1 is a distinct kind of cell death termed parthanatos (David et al., 2009[36]). Parthanatos is not caspase-dependent and is due to excess DNA damage and accumulation of poly(ADP) rubes (PAR) polymers, unlike apoptosis (Maru et al., 2023[100]). It accumulates the cell's NAD+ and ATP reserves, depleting them till an energy crisis and the cell's peculiar collapse (Covarrubias et al., 2021[31]). One of parthanatos main characteristics is the movement of AIF from the mitochondria to the nucleus, where it causes cell death (Huang et al., 2022[59]). This process is driven to a large extent by mitochondrial dysfunction, whereby excessive PAR polymer formation causes mitochondrial membrane potential loss, oxidative stress generation of ROS, and amplification of DNA impairment in a positive feedback loop to enhance PARP 1 activation (Ježek et al., 2018[63]). Parthanatos differs from necrosis in that it does not promote inflammatory cell swelling or apoptotic body formation, as occurs in apoptosis (Liu et al., 2022[88]). PARP-1 hyper activation, AIF translocation, and metabolic collapse make parthanatos a unique molecular signature unique to PARP-1 hyper activation and AIF translocation, and metabolic collapse is a very important cancer progression and therapy resistance process, as well as DNA damage in neurodegenerative disorders (Liu et al., 2022[92]). Multiple cancer models have been shown to respond to parthanatos induction, including in the context of oxidative stress and chemotherapeutic agents (An et al., 2024[4]).

**Figure 2:**
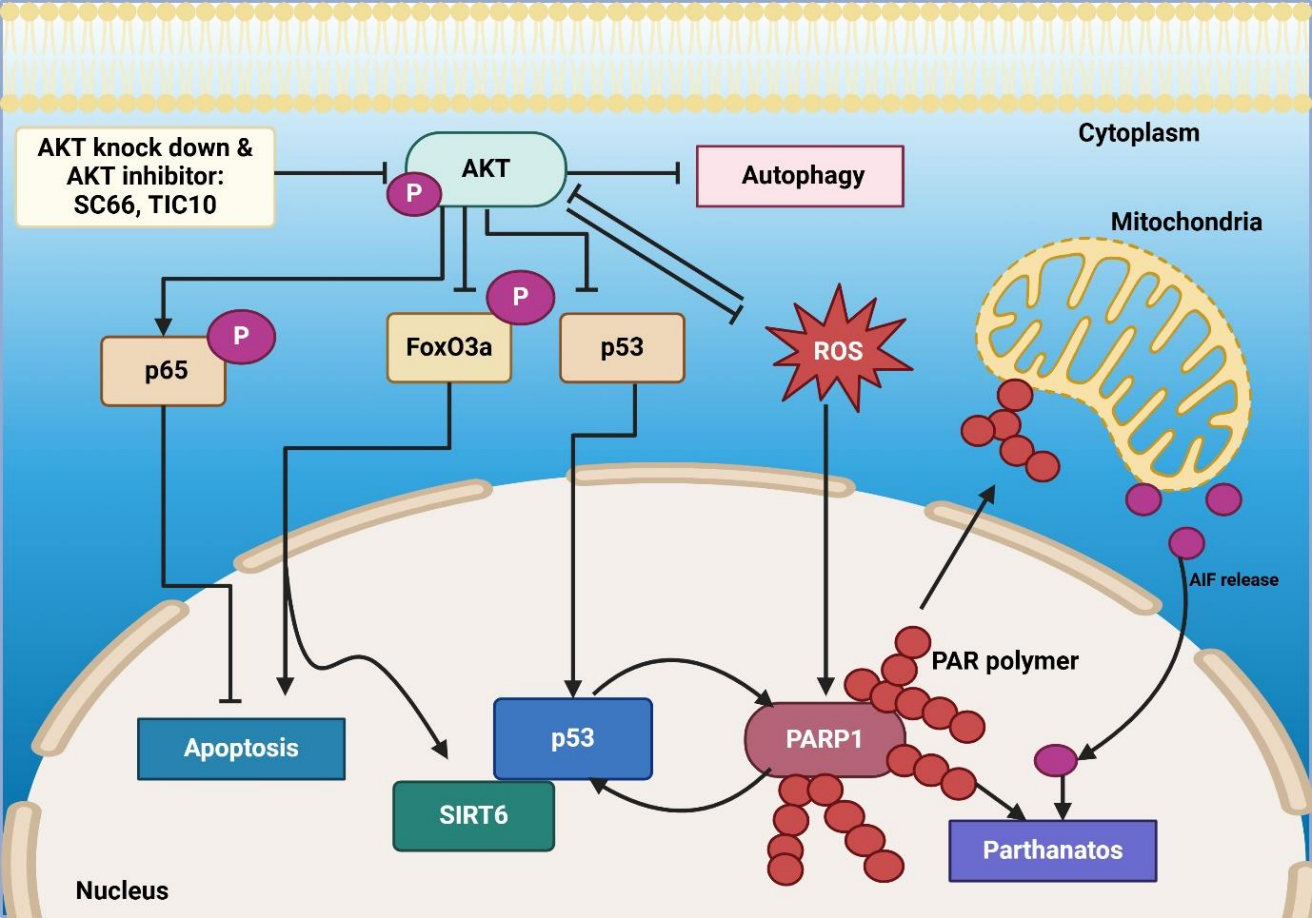
Figure 2 illustrates the regulation of apoptosis and parthanatos through AKT signaling, oxidative stress, and PARP1 activation. AKT promotes cell survival by inhibiting FoxO3a, p53, and autophagy while activating p65, which drives apoptosis. AKT knockdown or inhibition (SC66, TIC10) disrupts these pathways, enhancing p53 and FoxO3a activity. ROS accumulation induces PARP1 activation, leading to PAR polymer formation and mitochondrial AIF release, triggering parthanatos. SIRT6 modulates p53, linking apoptotic and parthanatos pathways, highlighting a complex interplay between survival and programmed cell death mechanisms.

Furthermore, in glioma cells, Ma et al. demonstrated that deoxypodophyllotoxin (DPT) caused parthanatos by the production of excessive ROS, the overexpression of PARP-1, the accumulation of PAR polymers, and the translocation of AIF into the nucleus. DPT-induced effects were reversed by knockdown of PARP-1, treatment with antioxidants (NAC), and PARP-1 inhibitors (3AB) rescuing cells (Ma et al., 2016[98]). Similarly, Zhang et al. identified a p53-SIRT6-PARP1 axis in colon cancer, where AKT inhibition promoted PARP-1 activation and parthanatos, whereas p53 deletion prevented its induction. However, AKT inhibition also led to protective autophagy, as shown in Figure 2(Fig. 2), suggesting that targeting both parthanatos and autophagy could improve therapeutic outcomes (Zhang et al., 2022[175]). 

Several studies have further linked the activation of parthanatos to small-molecule compounds with anticancer properties. The glycosylated flavonoid Brachydin A (BrA) was shown by Ribeiro et al. to cause parthanatos in metastatic prostate cancer spheroids by the disruption of mitochondrial membrane potential, activation of PARP-1, and nuclear translocation of AIF. The alteration of inflammatory indicators and anti/pro-apoptotic proteins was linked to BrA-mediated cell death (Ribeiro et al., 2022[123]). Similarly, Medrano et al. found that the cannabinoid derivative WIN-55 induced parthanatos in acute myeloid leukemia (AML) cells, evidenced by DNA damage, NAD+ depletion, and metabolic disruption. PARP inhibition with Olaparib prevented this effect, confirming that WIN-55-induced AML cell death depends on parthanatos (Medrano et al., 2024[103]). 

In addition to pharmacological triggers, environmental toxins and heavy metals have also been implicated in inducing parthanatos through oxidative stress and mitochondrial damage (Li et al., 2023[85]). Paul et al. demonstrated that fumonisin B1 (FB1), a mycotoxin, triggers parthanatos in neuroblastoma cells by causing excessive DNA damage, leading to PARP-1 overactivation, PAR accumulation, and AIF nuclear translocation. This process was further exacerbated by increased ROS generation, intracellular calcium accumulation, and endoplasmic reticulum stress, linking environmental toxins to parthanatos-mediated neurotoxicity (Paul et al., 2021[116]). In rat proximal tubular cells, Luo et al. investigated cadmium-induced parthanatos in more detail, showing that mitochondrial damage and oxidative stress cause PARP-1 activation and MAPK pathway participation, with JNK1/2 and p38 playing critical roles in apoptosis-parthanatos crosstalk (Luo et al., 2017[97]).

The molecular regulation of parthanatos has also been investigated in the context of AIF translocation and alternative cell death pathways (Tang et al., 2019[137]). Wang et al. explored the role of calpain in AIF release during parthanatos, demonstrating that AIF nuclear translocation occurs independently of calpain activation but can be prevented by PARP-1 inhibitors, suggesting that alternative mechanisms contribute to AIF release (Wang et al., 2009[152]). The clinical significance of parthanatos has been further validated in cancer specimens (Qiao et al., 2024[121]). Donizy et al. analyzed breast cancer tissues and found that high PARP-1 expression correlated with the absence of apoptotic bodies and necrosis, key cytomorphological features of parthanatos. Apoptotic bodies were connected to the redistribution PARP-1 from the nucleus to the cytoplasm, while necrosis was linked to decreased nuclear PARP-1 expression (Donizy et al., 2013[39]). These studies present strong evidence that parthanatos is a central mechanism of genome damage-induced cell death, oxidative stress, or metabolic disruption. Despite significant challenges related to apoptosis resistance, parthanatos treatment is possible through PARP inhibitors, ROS modulators, and oncogenic pathway regulators as a novel strategy to overcome this challenge. However, because parthanatos is both a tumor suppressor and tumor promoter, this mechanism remains a target for developing more effective and less mycotoxic therapeutic approaches (Figure 2(Fig. 2)).

### Role of parthanatos in cancer

In cancer, parthanatos acts as two mutually exclusive tumor-promoting and tumor-suppressing processes. The context of the cell determines whether parthanatos induces cancer or protects from it (Zhang et al., 2024[174]). Parthanatos is often brought about by extensive DNA damage through reactive oxygen species or chemotherapeutic agents that result in the overactivation of PARP-1, a hallmark of parthanatos (Chaitanya et al., 2010[19]). Parthanatos induction has emerged as a promising strategy in cancer therapy to eliminate tumor cells, particularly apoptosis-resistant ones, as in cancer treatment (Lou et al., 2020[94]). Excessive PARP-1 activation is commonly required for parthanatos downstream to such DNA-damaging agents as platinum compounds and radiation therapy. It is causative of cell death through mitochondrial dysfunction, AIF translocation, and apoptosis-inducing factor-dependent cell death (Ke et al., 2019[67]). Also, synthetic lethality for tumors with defective DNA repair involves PARP inhibitors that leverage synthetic lethality to induce parthanatos selectively in DNA repair deficient tumors (Helleday, 2011[57]). Although parthanatos also aids in tumor progression and therapy resistance, it also plays a role in autophagy (Yang et al., 2024[169]). Hyperactivation of PARP-1 in the tumor microenvironment may be chronic, leading to genomic instability and inflammatory signaling for tumorigenesis and metastasis (Chen et al., 2022[24]). In addition, cancer cells develop adaptive mechanisms to resist parthanatos, allowing themselves to survive in oxidative stress or conditions of DNA damage (An et al., 2024[4]). Zhao et al. studied the impact of survivin suppressant sepantronium bromide (YM155) on ESCC. They found that YM155 effectively inhibited tumor development by lowering survivin levels and preventing cell death by genetically knocking down AIF or PARP-1 (Zhao et al., 2015[176]). Similarly, Boulos et al. demonstrated that cynaropicrin, a sesquiterpene lactone, induces parthanatos in hematopoietic tumors, including multiple myeloma and leukemia. In a zebrafish xenograft model of T-cell acute lymphoblastic leukemia, cyanaropicrin-mediated PARP-1 hyperactivation resulted in PAR polymer buildup and AIF nuclear translocation, successfully inhibiting tumor development (Boulos et al., 2023[14]). Zheng et al. examined the parthanatos in glioma cells due to oxidative stress. They found that exposure to H₂O₂ led to ROS accumulation, PARP-1 activation, AIF nuclear translocation, and mitochondrial depolarization. Inhibiting JNK suppressed ROS production, suggesting potential therapeutic agents (Zheng et al., 2017[177]). Similarly, in OSCC, Li et al. found that the chemotherapeutic drug oxaliplatin causes parthanatos by causing nuclear translocation of AIF, depolarization of the mitochondrial membrane, and overactivation of PARP-1. Oxaliplatin-induced parthanatos was prevented by treatment with PARP-1 inhibitors or antioxidants, demonstrating the role of oxidative stress in inducing this kind of cell death (Li et al., 2021[84]).

While parthanatos serves as an important cell death mechanism, chronic PARP-1 hyperactivation may paradoxically promote therapy resistance and tumor progression (Lee et al., 2018[80]). Liu et al. identified a novel mechanism by which cyclophosphamide induces parthanatos in leukemia cells through glutathione peroxidase 4 (GPX4) degradation. This process led to AIF nuclear translocation but not ferroptosis, demonstrating an alternative pathway by which leukemia cells may evade apoptosis. Knockdown of AIFM1 or overexpression of GPX4 reversed cyclophosphamide-induced cell death, suggesting that parthanatos modulation could impact therapy resistance (Liu et al., 2022[90]). Zhou et al. further explored the role of parthanatos in leukemia therapy using palladium (II) complexes (J4 and J6). These compounds selectively induced PARP-1 hyperactivation and AIF nuclear translocation, overcoming apoptosis resistance in leukemia cells while sparing normal cells. Interestingly, J4 and J6 did not significantly induce apoptosis or necrosis, reinforcing the specificity of parthanatos induction as a cytotoxic strategy (Zhou et al., 2023[180]).

Parthanatos has also been implicated in inflammatory diseases, reinforcing its relevance beyond oncology (Vitale et al., 2023[143]). Xu et al. demonstrated that necrotizing enterocolitis (NEC)--affected intestinal tissues exhibit elevated parthanatos markers, including AIF nuclear translocation, PAR polymer accumulation, and PARP-1 activation. Inhibition of PARP-1, either pharmacologically (3-AB) or genetically (Parp1 knockout), significantly reduced intestinal injury and inflammation, suggesting that targeting parthanatos may mitigate NEC-related epithelial damage (Xu et al., 2024[164]). Similarly, Xue et al. identified a novel role for parthanatos in macrophage cell death during sepsis. The study linked TLR4 upregulation to ROS-induced PARP-1 activation, leading to AIF nuclear translocation. Pharmacological inhibition of TLR4 signaling prevented parthanatos in macrophages, revealing a potential target for reducing endotoxemia-related inflammatory damage (Xue et al., 2021[167]). These studies also show that parthanatos, while aborted, is a highly context-dependent form of cell death with both therapeutic and pyknic advantages. Inducing parthanatos in apoptosis-resistant tumors is a promising strategy, but chronic PARP-1 hyperactivation can drive genomic instability and proinflammatory signaling, resulting in tumor progression and therapy resistance (Table 1(Tab. 1); References in Table 1: Liu et al., 2022[91]; Luo et al., 2017[97]; Ma et al., 2016[98]; Medrano et al., 2024[103]; Mo et al., 2022[107]; Paul et al., 2021[116]; Ribeiro et al., 2022[123]; Xu et al., 2024[164]; Xue et al., 2021[167]; Zhao et al., 2015[176]; Zhou et al., 2023[180]).

## Apoptosis vs. Parthanatos in Cancer

### Distinct molecular and morphological features

Apoptosis and parthanatos are two definable programmed cell deaths with allowable substrate and morphology specificity (Cao et al., 2024[17]). Apoptosis, usually called type I, is a caspase-dependent process involving chromatin condensation, DNA fragmentation into nucleosome-sized fragments, membrane blebbing, and the release of apoptotic bodies (Kari et al., 2022[66]). If engulfed by neighboring or immune cells, these bodies are resolved non-inflammatory (Szondy et al., 2017[136]). Intrinsic and extrinsic pathways tightly control the process, and their key players are caspases, cytochrome c, and Bcl-2 family proteins (Singh et al., 2019[132]). It is a highly controlled mechanism that does not cause excessive tissue damage and inflammatory responses (Chen et al., 2018[23]). Severe damage to DNA and oxidative stress can cause parthanatos, a caspase-independent cell death triggered by PARP 1 (Gao et al., 2023[48]). In terms of appearance, parthanatos differs from necrosis and pyknosis in that it exhibits chromatin condensation, large-scale DNA fragmentation, and the absence of apoptotic structures or cellular swelling, which are hallmarks of necrosis (Tang et al., 2019[137]). Parthanatos is characterized by the translocation of AIF from mitochondria to the nucleus, resulting in extensive DNA breakage (Mashimo et al., 2013[101]).

In contrast to apoptosis, a cellular collapse that results from parthanatos is due to the hyperactivation of PARP-1, resulting in the depletion of cellular energy stores (NAD+ and ATP). Biologically, there is a distinction between the underlying means of cell death for these two mechanisms, as some cancer cells suppress apoptosis but remain a target of parthanatos (Martínez-Morcillo et al., 2021[99]). Cheong et al. examined the role of CASK in microglial parthanatos triggered by H₂O₂ exposure in CHME3 cells. Silencing of CASK protected cells from oxidative stress-induced parthanatos by reducing PARP-1 activation, mitochondrial dysfunction, and reactive oxygen species (ROS) production, preventing mitochondrial fission and enhancing oxidative phosphorylation. Interestingly, no AIF translocation indicated a non-canonical AIF-independent form of parthanatos. CASK silencing also altered AKT and AMPK signaling, suggesting its potential as a therapeutic target for central nervous system disorders involving oxidative stress and parthanatos (Cheong et al., 2024[27]). Kuzhandaivel et al. investigated kainate-induced excitotoxicity in neonatal rat spinal cord neurons, revealing that parthanatos was the primary neuronal death mechanism. Strong PARP-1 activation, poly(ADP-ribose) production, and AIF nuclear translocation were observed, while caspase-3 inhibition failed to prevent neuronal damage, distinguishing parthanatos from apoptosis. Notably, pharmacological inhibition of PARP-1 reduced neuronal death, highlighting PARP-1 as a key therapeutic target in acute spinal cord injury (Kuzhandaivel et al., 2010[78]).

Studies examining harsh environmental circumstances provide more evidence of parthanatos' involvement in stress-induced cell death (Wang and Ge, 2020[149]). Schiefer et al. analyzed heat-induced cell death in human fibroblasts, revealing that hyperthermia primarily triggers parthanatos rather than apoptosis. Heat exposure significantly increased PARP-1 protein expression, DNA double-strand breaks, ATP depletion, and AIF nuclear translocation, with no evidence of caspase activation or apoptotic bodies. The temporal sequence of parthanatos induction, with a peak at five hours post-heat exposure, underscores its delayed onset compared to apoptosis. These findings provide valuable insights into burn injury pathophysiology and the role of parthanatos in thermal stress responses (Schiefer et al., 2024[127]).

The ability of cancer cells to evade apoptosis while maintaining susceptibility to parthanatos has been explored in multiple models (Wang et al., 2022[146]). Nuclear Ca^2+-^dependent endonucleases and CPP32/Yama protease activity, two essential apoptosis executioners, are markedly reduced in highly metastatic cells, according to research by Glinsky et al. on apoptosis resistance in metastatic cancer cell lines. According to these results, metastatic cells create defenses against apoptosis, allowing them to continue growing and surviving (Glinsky et al., 1997[51]). Similarly, Baile et al. reported that in glioblastoma, cell death followed a caspase-dependent apoptotic pathway, whereas in pancreatic and colorectal cancer cells, it involved programmed necrosis. The latter was characterized by mitochondrial membrane depolarization, AIF nuclear translocation, and PARP-1 activation, hallmarks of parthanatos. Resistant cell lines exhibited enhanced detoxification mechanisms, emphasizing the variability in cell death pathways across different cancer types (Fuentes-Baile et al., 2020[44]).

Additional research has highlighted the dose-dependent nature of programmed cell death mechanisms (Kopeina and Zhivotovsky, 2022[74]). Lennon et al. found that lower doses of toxic agents-initiated apoptosis, characterized by an internally programmed death process, while higher doses led to necrosis due to irreversible membrane failure. Furthermore, extracellular calcium was essential for apoptosis initiation, underscoring the role of calcium homeostasis in determining cell fate (Lennon et al., 1991[81]). Fujikawa et al. reported that neuronal damage was characterized by nuclear pyknosis, chromatin condensation, cytoplasmic vacuolation, and mitochondrial swelling. Although some neurons exhibited DNA fragmentation, this was associated with necrosis rather than classical apoptosis, further supporting the idea that programmed cell death mechanisms can be context-dependent (Fujikawa et al., 2000[45]). These studies emphasize molecular and morphological differences between apoptosis and parthanatos. Whereas apoptosis proceeds through a caspase-dependent, energy-sufficient pathway characterized by the fragmentation of the cell into apoptotic bodies, parthanatos is executed by PARP-1 hyperactivation, AIF translocation to the nucleus, and subsequent massive degradation of DNA. Parthanatos differs from apoptosis and necrosis because it does not initiate apoptotic body formation and depletes cellular energy. The mechanistic differences between these cell death pathways are critical to understanding since these results have broad consequences for intervening therapeutically in cancer, neurodegeneration, and inflammatory diseases. 

### Overlapping pathways and interplay

The programmed cell death processes of apoptosis and parthanatos share several regulatory pathways despite being different mechanisms in terms of their cellular destruction operations (Kist and Vucic, 2021[71]). Mutually exclusive patterns of cell death initiate due to extreme cellular pressure, such as DNA damage alongside oxidative stress or genotoxic elements, while depending on pathological mitochondrial problems as their foundation (Shimura, 2023[128]). The apoptotic cascade triggers cytochrome c release from mitochondria to activate caspases, but parthanatos makes mitochondria release AIF that moves to the nucleus for extensive DNA fragmentation (Nguyen et al., 2023[113]). These two distinct cellular death pathways listen to ROS and DNA damage signals, yet the degree of cellular stress and its duration guide which form of cell death will occur (Redza-Dutordoir and Averill-Bates, 2016[122]). The cellular pathways display communication through Bid, which enhances parthanatos by increasing membrane permeability in mitochondria (Chipuk et al., 2021[29]).

On the other hand, if caspases are inhibited or impaired, cells may revert to parthanatos as a second line of death (Yan et al., 2020[168]). Jiang et al. studied oxidative stress-induced cell death in stria marginal cells using a glucose oxidase/glucose model. They found that GO/G triggers parthanatos, which can be rescued by knocking down PARP-1, and autophagy plays a pro-survival role (Jiang et al., 2018[64]). Similarly, Chiu et al. studied MNNG-induced parthanatos in MEFs, revealing it triggers rapid ROS production, JNK activation, PARP-1 activation, and intracellular calcium elevation, sustaining DNA damage and PARP-1 activation (Chiu et al., 2011[30]).

The function of parthanatos in cancer cell death regulation has been further explored regarding p53 status and autophagy (Zhang et al., 2022[175]). So et al. found that sodium arsenite treatment induced parthanatos in p53-deficient H1299 cells, with inhibition of autophagy enhancing parthanatos and p53 overexpression reducing it, suggesting targeting PARP-1 could be a potential strategy (So and Oh, 2023[133]). Zhu et al. found that Stanniocalcin-1 (STC1) is upregulated in colitis models and Crohn's disease patients, causing increased parthanatos and inflammation. Knockout of STC1 reduced inflammation and disease severity. STC1 interacts with PARP-1 and activates the JNK pathway, suggesting the STC1-PARP1-JNK axis as a potential therapeutic target (Zhu et al., 2024[182]).

The interaction between apoptosis and parthanatos is also evident in tumor repopulation mechanisms (Shoshan-Barmatz et al., 2023[130]). Huang et al. demonstrated that caspase-3 activation in apoptotic tumor cells paradoxically promotes tumor regrowth during radiotherapy by stimulating prostaglandin E2 (PGE2) production. Caspase-3-deficient tumors exhibited enhanced sensitivity to radiotherapy, while human cancer tissues with higher caspase-3 activation levels correlated with increased recurrence and mortality rates. These findings highlight a paradoxical role of apoptosis in therapy resistance and tumor survival, suggesting that blocking caspase-3 activation could improve radiotherapy outcomes (Huang et al., 2011[60]). Melcher et al. further explored the immunogenic potential of different cell death modalities, revealing that apoptotic colorectal cancer cells exhibited lower immunogenicity due to reduced HSP expression, whereas non-apoptotic forms of cell death induced stronger immune responses. Overexpression of Bcl-2 inhibited apoptosis and increased HSP levels, and transfection with hsp70 further enhanced the immunogenicity of tumor cells, suggesting that promoting non-apoptotic cell death pathways may improve cancer immunetherapy (Melcher et al., 1998[104]).

Additional studies have identified novel molecular mechanisms linking apoptosis and immunogenic cell death (ICD) (Chang et al., 2023[21]). Panaretakis et al. found that pre-apoptotic exposure of calreticulin and ERp57, crucial markers of ICD, requires ER stress-induced activation of the PERK pathway, partial caspase-8 activation, and BAP31 cleavage. Depletion of SNAREs, caspase-8, or PERK abolished CRT/ERp57 exposure without affecting cell death, suggesting that these factors are critical for the pre-apoptotic exposure to calreticulin but do not directly influence the execution of cell death (Panaretakis et al., 2009[114]). Furthermore, Xia et al. created a predictive model for ovarian cancer prognosis using 12 PCD patterns and identified a 20-gene set. They found high-risk patients' sensitivity to dasatinib and correlations with immune checkpoint genes and tumor microenvironment components, aiding in targeted therapy (Xiao et al., 2023[162]). Collectively, these findings emphasize the complex interrelationship between apoptosis and parthanatos, their shared regulatory networks, and their therapeutic implications. Though apoptosis as a tumor suppressive mechanism is well established, the activation of apoptosis sometimes proves harmful to the tumor by facilitating immune evasion and repopulation. On the contrary, parthanatos represents an alternative cell death mechanism and can be used to overcome apoptosis resistance in therapy-resistant cancer cells (Table 2(Tab. 2); References in Table 2: Cheong et al., 2024[27]; Chiu et al., 2011[30]; Fuentes-Baile et al., 2020[44]; Fujikawa et al., 2000[45]; Glinsky et al., 1997[51]; Huang et al., 2011[60]; Jiang et al., 2018[64]; Kuzhandaivel et al., 2010[78]; Lennon et al., 1991[81]; Melcher et al., 1998[104]; Panaretakis et al., 2009[114]; Schiefer et al., 2024[127]; So and Oh, 2023[133]; Wang et al., 2024[145]; Zhu et al., 2024[182]). 

## Therapeutic Targeting of Cell Death Pathways

### Strategies targeting apoptosis

Cancer therapy development has focused on apoptosis since researchers seek to recover cancer cells' ability to undergo programmed death (Mirzayans, 2024[106]). Cancer cells avoid programmed cell death by producing Mcl-1, Bcl-xL, and Bcl-2, and genetic mutations cause alterations in pro-apoptotic control, such as TP53 (Akl et al., 2014[1]). Developers have established several approaches to restore apoptotic signaling pathways against the mechanisms that prevent it (Indran et al., 2011[61]). Venetoclax is a BH3 mimetic drug that stops anti-apoptotic Bcl-2 proteins from functioning so that pro-apoptotic Bax and Bak activation can begin the apoptosis sequence (Roberts and Huang, 2017[124]). The clinical benefits of death receptor agonists working through TRAIL receptors to activate extrinsic apoptosis fail to produce desirable results (Kundu et al., 2022[76]). Small molecules, called SMAC mimetics, remove the inhibitory function of apoptosis proteins (IAPs), so tumors become more apoptotically sensitive (Bai et al., 2014[8]). Studies in recent years have evaluated different chemical approaches and molecular methods for cancer apoptosis regulation (Hassan et al., 2014[55]). Gali-Muhtasib et al. examined thymoquinone (TQ), which originates from Nigella sativa as an active substance to study its cancer-fighting properties and cell apoptotic effects on HCT-116 colon cancer cells. The dosage and time duration determined how TQ caused arrest in G1 phase along with apoptosis in HCT-116 cells. The increase of p53 and p21WAF1 expression together with Bcl-2 level decrease corresponded to apoptosis development yet p53 inhibition through pifithrin-α blocked these effects. TQ demonstrated resistance to p53-null cells indicating that apoptosis derived from TQ treatment relies on p53 (Gali-Muhtasib et al., 2004[47]). Lucena et al. evaluated the cytotoxic and anti-inflammatory effects of 5-fluorouracil (5-FU) incorporated into Cu-BTC metal-organic frameworks (MOFs). Structural characterization confirmed stable drug loading, with a sustained release profile reaching 82 % in 48 hours. Cytotoxicity assays revealed significant apoptosis induction in MCF-7 breast cancer and HL60 leukemia cells. Additionally, the formulation reduced leukocyte counts and inflammatory cytokines in rodent models, highlighting its potential as a sustained-release chemotherapeutic agent with anti-inflammatory benefits (Lucena et al., 2013[95]).

The role of IAP proteins as therapeutic targets has also been extensively investigated (Vucic and Fairbrother, 2007[144]). Fulda demonstrated that IAP proteins regulate survival and cell death by controlling caspase activation and NF-κB signaling. Since many human cancers exhibit aberrant IAP overexpression, small-molecule IAP antagonists have been developed to mimic SMAC and induce tumor cell death or sensitize tumors to cytotoxic therapies. Several IAP antagonists, including GDC-0917, LCL161, and AT-406, are undergoing clinical trials, underscoring their potential in combination therapies (Fulda, 2014[46]). Chen et al. explored a dual-targeting strategy for inducing mitochondria-dependent apoptosis using a functionalized pro-apoptotic peptide conjugated with folic acid (FA) for receptor-mediated endocytosis and triphenylphosphonium (TPP) cation for mitochondrial localization. This system efficiently targeted cancer cells, causing severe mitochondrial damage, dysfunction, and subsequent apoptosis, demonstrating its potential as a precise cancer therapeutic (Chen et al., 2013[26]).

Apoptosis-inducing strategies have also been linked to oxidative stress modulation (Chandra et al., 2000[20]). Liu et al. studied Adpa-Mn, a novel manganese compound, for its anticancer activity. They found it selectively inhibited cancer cell growth through the TfR system, induced apoptosis and autophagy, and reduced its cytotoxic effects with antioxidant treatment (Liu et al., 2015[87]). Becattini et al. identified Bid as a promising therapeutic target for modulating apoptosis in neurodegeneration and ischemia. Using the SAR by ILOEs approach, researchers developed small-molecule inhibitors that bind Bid and prevent its activation, inhibiting SMAC release and caspase-3 activation. This represents the first known strategy targeting a pro-apoptotic Bcl-2 protein for therapeutic intervention (Becattini et al., 2004[11]).

Emerging approaches also explore combination therapies integrating PDT, PTT, and immunotherapy (Kong and Chen, 2022[73]). Li et al. (2019[86]) developed a double endoplasmic reticulum-targeting nanosystem that combines PDT, PTT, and immunotherapy for enhanced tumor eradication. The system uses ER-targeting peptides conjugated with indocyanine green-coated hollow gold nanospheres and oxygen-delivering liposomes to induce ROS-mediated ER stress, triggering immunogenic cell death (ICD) and immune stimulation (Li et al., 2019[86]).

The therapeutic potential of apoptosis-targeting drugs has also been demonstrated in HNSCC (Axelrod et al., 2015[6]). Zhang et al. investigated the effects of sepantronium bromide (YM155) in HNSCC, showing that YM155 suppresses survivin expression, induces apoptosis via mitochondrial and death receptor pathways, and enhances autophagy through Beclin1 upregulation. YM155 also inhibits the mTOR signaling pathway, enhancing tumor cell death. In HNSCC xenograft models, YM155 significantly delayed tumor onset, suppressed tumor growth and exhibited synergy with docetaxel without notable toxicity. These findings support the clinical potential of YM155 as a targeted therapy for HNSCC (Zhang et al., 2015[173]). This research presents various methods for restoring cancer cell apoptosis through small-molecule protein inhibitors, optimal drug delivery methods, and combined treatment approaches. Proper cancer treatment necessitates apoptosis induction, but new methodologies that combine oxidative stress regulation with immunogenic cell death and site-specific drug delivery will likely create advanced solutions. Future investigations must expand their effort toward developing optimized combination therapies that make cancer cells more sensitive to apoptosis while decreasing resistance factors to achieve better clinical results in refractory and aggressive cancer cases.

### Innovations in parthanatos modulation

Pioneering drug treatments targeted at parthanatos represent promising therapeutic options mainly used to address cancers resistant to conventional apoptosis-based treatments (D'Amico and De Amicis, 2024[33]). Efforts to control PARP-1 have become important because it functions as the main driver of parthanatos (Wang et al., 2011[153]). PARP inhibitors, including olaparib and rucaparib, are therapeutic agents that use synthetic lethality within DNA repair-deficient tumors with BRCA mutations (Kim and Nam, 2022[69]). Scientists are investigating new approaches to enhance the parthanatos pathway activity (Wu et al., 2023[157]). Combining reactive oxygen species (ROS) enhancement agents or direct mitochondrial dysfunction inducers with PARP activators creates more potent than additive effects for parthanatos development (Yu et al., 2022[170]). Researchers use AIF nuclear translocation inhibitors to explore ways of optimizing treatment effects and reducing adverse side effects (Doti et al., 2014[40]). Research conducted recently has expanded knowledge about how to modify parthanatic death in cancer therapy and various pathological settings (Liu et al., 2022[88]). Zhou et al. identified ZINC253504760, a synthetic cardenolide, as a novel anticancer agent capable of inducing parthanatos in multidrug-resistant (MDR) leukemia cells. The compound effectively induced G2/M phase arrest and triggered a distinct form of cell death marked by PARP overactivation, AIF nuclear translocation, DNA damage, and mitochondrial membrane collapse. Interestingly, parthanatos induction by ZINC253504760 was ROS-independent, suggesting a unique mechanism of action. Additionally, the compound acted as an ATP-competitive MEK inhibitor, positioning it as a dual-function agent for overcoming MDR in leukemia (Zhou et al., 2023[179]). Han et al. explored the role of parthanatos in subarachnoid hemorrhage (SAH) and demonstrated that the PARP-1 inhibitor AG14361 significantly reduced oxidative stress, inflammation, and neuronal apoptosis in SAH models. Treatment with AG14361 improved neurological function and blood-brain barrier integrity by suppressing parthanatos, highlighting the potential of PARP-1 inhibitors beyond oncology in neuroprotective therapies (Han et al., 2024[54]).

**Figure 3:**
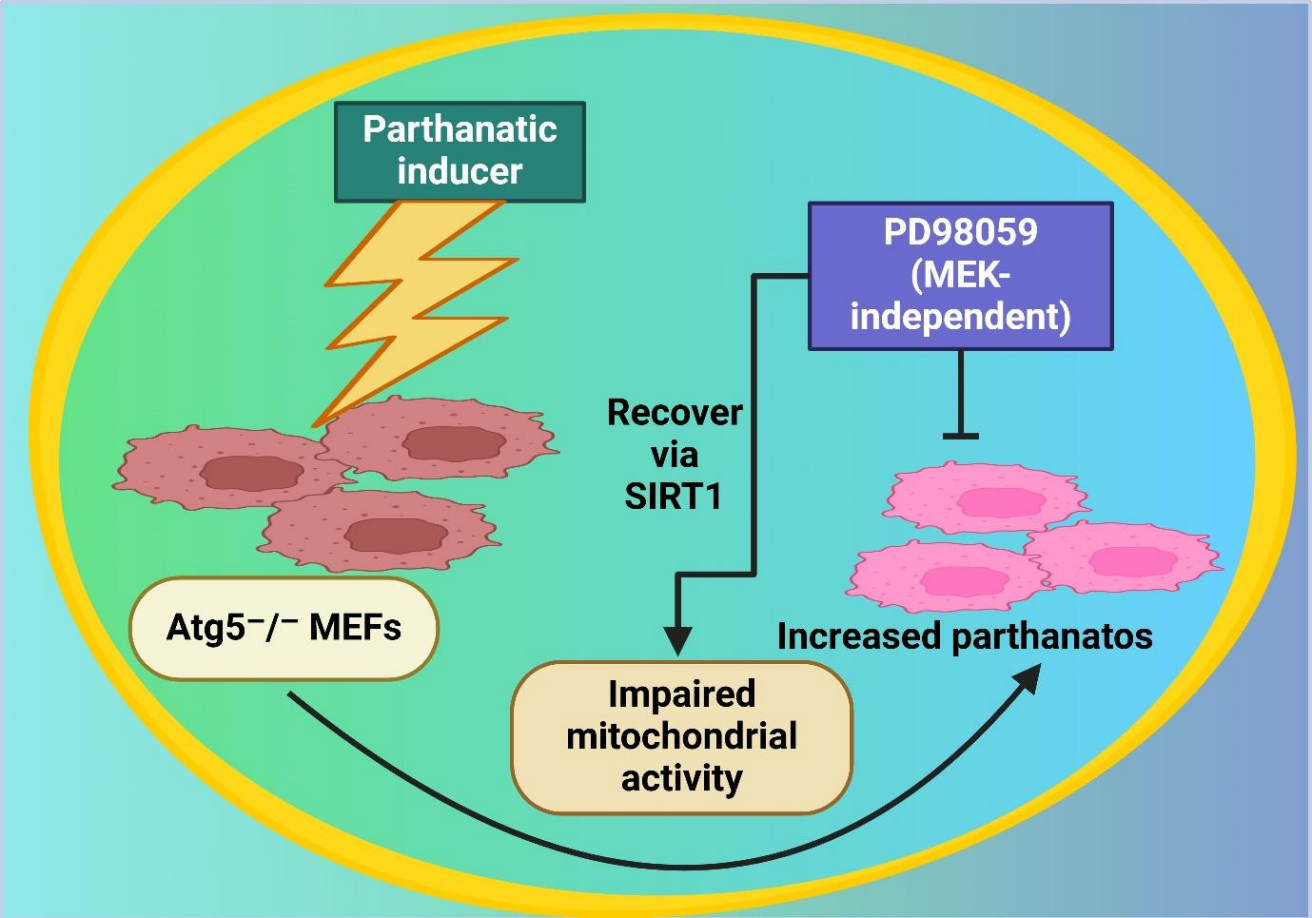
Figure 3 illustrates the regulation of parthanatos and mitochondrial activity in Atg5⁻/⁻ mouse embryonic fibroblasts (MEFs). A parthanatic inducer triggers cell death, leading to impaired mitochondrial activity. SIRT1 facilitates recovery from mitochondrial dysfunction. However, treatment with PD98059, a MEK-independent inhibitor, enhances parthanatos, suggesting its role in promoting cell death. The interplay between autophagy deficiency, mitochondrial impairment, and SIRT1-mediated recovery highlights the complex regulatory mechanisms of parthanatos in cellular stress responses.

The bioenergetic regulation of parthanatos has also been investigated in the context of metabolic stress (Huang et al., 2014[50]). Huang et al. examined how mitochondrial dysfunction contributes to parthanatos in Atg5-deficient cells. The study found that the parthanatic inducer MNNG triggered ATP depletion in wild-type (WT) mouse embryonic fibroblasts (MEFs), but Atg5-deficient MEFs exhibited impaired energy restoration due to reduced SIRT1 activity and mitochondrial dysfunction. Interestingly, treatment with PD98059 enhanced mitochondrial activity and ATP restoration during parthanatos without interfering with DNA damage responses, as shown in Figure 3(Fig. 3). These findings suggest energy-based therapeutic strategies for mitigating parthanatos-related disorders (Huang et al., 2014[58]). Wang et al. further investigated the molecular regulation of AIF nuclear translocation in parthanatos and identified AIF as a high-affinity poly(ADP-ribose) (PAR)-binding protein. PAR binding facilitated AIF release from mitochondria and its nuclear translocation, which was essential for PARP-1-mediated cell death. Mutation of AIF's PAR-binding site prevented its release and nuclear translocation while preserving its NADH oxidase function. These findings highlight the PAR-AIF interaction as a potential target for modulating parthanatos (Wang et al., 2011[153]).

Emerging evidence suggests that parthanatos may contribute to reproductive aging and ovarian dysfunction (Chiang et al., 2020[28]). Batnasan et al. demonstrated that cumulus granulosa cells (GCs) from patients with diminished ovarian reserve (DOR) exhibited increased PARP-1 activation and AIF nuclear translocation, indicative of parthanatos-mediated cell death. Using an in vitro model of H₂O₂-induced parthanatos in ovarian cells, the study showed that melatonin inhibited PARP-1 activation, reduced AIF translocation, and mitigated oxidative stress-induced parthanatos. These findings suggest that melatonin may protect against ovarian aging and oxidative damage (Batnasan et al., 2020[10]).

The role of parthanatos in chemotherapeutic responses has also been explored in leukemia models (Messikommer et al., 2021[105]). Maru et al. demonstrated that cytarabine and idarubicin induced parthanatos in acute myeloid leukemia (AML) cells, particularly in M4/M5 subtypes. Parthanatos induction was observed in 30 % of AML cell lines and 46 % of AML patient samples, with patients exhibiting parthanatos showing significantly improved survival rates. Furthermore, optimal PARP-1 expression levels correlated with drug sensitivity, whereas overexpression or suppression reduced chemotherapy efficacy. These findings highlight parthanatos as a crucial determinant of treatment response in AML (Maru et al., 2023[100]).

Mitochondrial dysfunction and oxidative stress are key drivers of parthanatos in gliomas (Liu et al., 2022[91]). Wang et al. investigated the role of Tax1 binding protein 1 (TAX1BP1) in deoxypodophyllotoxin (DPT)-induced parthanatos in glioma cells. The study found that DPT treatment led to excessive ROS production, PARP-1 overactivation, and mitochondrial TAX1BP1 redistribution, facilitating AIF nuclear translocation. TAX1BP1 enhanced respiratory chain complex I activity by upregulating ND1, ND2, NDUFS2, and NDUFS4, leading to superoxide generation, mitochondrial depolarization, and DNA damage. DPT treatment in U87 xenografts suppressed tumor growth via NAD+ depletion and parthanatos induction, identifying TAX1BP1 as a key mediator of parthanatos in gliomas (Wang et al., 2023[150]).

In addition to targeting parthanatos, novel platinum-based drugs have been developed to bypass apoptosis resistance (Al-Khayal et al., 2020[2]). Guo et al. introduced Mono-Pt, a monofunctional platinum(II) complex that induces autophagic cell death in apoptosis-resistant ovarian cancer cells. Unlike cisplatin, which primarily induces apoptosis, Mono-Pt triggered cytoplasmic vacuolation, LC3-II accumulation, and autophagic vacuole formation, dependent on BECN1 and ATG7 expression. The study further demonstrated that Mono-Pt's effects were mediated through the AKT1-MTOR and MAPK1/3 pathways, offering a potential alternative for overcoming chemotherapy resistance in ovarian cancer (Guo et al., 2013[53]). The research confirms that parthanatos modulation strategies show promise for treating cancer and other medical conditions. PARP inhibitors continue as central parthanatos-targeted treatments, but future therapeutic developments include enhancements of reactive oxygen species production, mitochondrial control adaptations, and AIF transaction regulations. The clinical importance of parthanatos expands into neuroprotection, reproductive aging, immune modulation, and its relevance in oncology research (Figure 3(Fig. 3)).

### Dual-pathway targeting

The research confirms that parthanatos modulation strategies show promise for treating cancer and other medical conditions (Damiescu et al., 2024[34]). The discovery of PARP inhibitors is essential in parthanatos-targeting methods, but investigators have identified three new therapeutic strategies involving ROS enhancement and mitochondrial control alongside AIF regulation mechanisms (Koehler et al., 2021[72]). The clinical importance of parthanatos expands into neuroprotection, reproductive aging, immune modulation, and its relevance in oncology research (Chen et al., 2024[22]). Dual-pathway targeting gives effective results during the treatment of tumors that exhibit resistance to apoptosis through p53 or caspase dysfunction (Hassin and Oren, 2023[56]). The combination therapy shows promise for handling aggressive tumors and therapy-resistant cancer types (Bahreyni et al., 2024[7]).

Numerous research evaluations confirm that targeting two protective pathways yields successful results when treating cancer (Kelley et al., 2014[68]). Wen et al. investigated arnicolide D, a compound derived from *Centipede minima*, and its anti-tumor effects in breast cancer cells. Arnicolide D induced oxidative stress, increasing ROS levels, mitochondrial dysfunction, and LDH release while significantly reducing cell viability. Notably, it activated multiple cell death pathways, including apoptosis, parthanatos (via PARP-1 activation and AIF nuclear translocation), and ferroptosis (via GPX4 suppression and Fe²⁺/ MDA accumulation). Additionally, arnicolide D inhibited cell invasion by downregulating MMP-2 and MMP-9, highlighting its potential as a multi-target therapeutic agent (Wen et al., 2024[154]). Li et al. further explored ROS-inducing agents in cervical cancer and found that massive DNA strand breaks caused by excessive 8-oxoG excision by OGG1 led to parthanatos induction. Inhibition of MTH1 enhanced this effect by increasing 8-oxoG incorporation into DNA, creating a synergistic effect with ROS-inducing agents. *In vivo*, this combination significantly suppressed tumor xenograft growth, particularly in OGG1-proficient models, demonstrating a selective and safer chemotherapeutic strategy (Li et al., 2024[83]).

Dual-pathway targeting has also been explored in apoptosis-resistant tumors (Fitzgerald et al., 2022[43]). Xavier et al. demonstrated that ursolic acid (UA) effectively induced caspase-independent apoptosis in p53-mutant HCT15 CRC cells while enhancing the effects of 5-FU via JNK pathway activation. UA also modulated autophagy, as indicated by increased LC3 and p62 expression, contributing to JNK-dependent cell death. *In vivo*, UA significantly reduced tumor growth in HCT15 xenograft models, underscoring its potential to overcome chemotherapy resistance (Xavier et al., 2013[161]). Similarly, Mujumdar et al. showed that triptolide induces caspase-dependent apoptosis in some pancreatic cancer cells and CICD in metastatic lines. Blocking autophagy shifted cell death to apoptosis while inhibiting both pathways rescued cells, highlighting triptolide's dual mechanism as a therapeutic strategy (Mujumdar et al., 2010[109]).

The interplay between apoptosis and autophagy has also been investigated in prostate and breast cancers (Bhutia et al., 2010[12]). In MCF-7 breast cancer cells, resveratrol causes both caspase-dependent and independent cell death, according to research by Scarlatti et al. It causes Beclin 1-independent autophagy in cells lacking caspase-3, while it causes both autophagy and apoptosis in cells expressing caspase-3. This supports the therapeutic potential of resveratrol by indicating that its induction of autophagy compensates for a lack of apoptosis (Scarlatti et al., 2008[126]). Kim et al. discovered that geraniol suppresses mTOR by blocking AKT and activating AMPK, which causes PC-3 prostate cancer cells to undergo apoptosis and autophagy. Cell mortality was increased by co-treatment with an AMPK activator and an AKT inhibitor, indicating a dual-pathway approach to prostate cancer treatment (Kim et al., 2012[70]).

Oxidative stress modulation has become crucial in dual-pathway targeting (Liu et al., 2022[90]). According to Wu et al., ginsenoside Rh4 inhibits the development of colorectal cancer by triggering autophagy, caspase-dependent apoptosis, and G0/G1 arrest. ROS buildup triggers apoptosis by activating the JNK-p53 pathway. Cell mortality was decreased by blocking ROS, JNK, or p53, demonstrating Rh4's potential as an anticancer drug that targets oxidative stress (Wu et al., 2018[158]). Wang et al. demonstrated that co-treatment of curcumin and berberine suppresses the development of breast cancer cells by causing autophagic cell death via the JNK/Bcl-2/Beclin1 pathway and caspase-dependent apoptosis via ERK. JNK inhibition decreased cytotoxicity and autophagy indicators, demonstrating the combination's therapeutic potential (Wang et al., 2016[147]). Combining targeted strategies toward two cell death pathways enhances cancer treatment quality by unifying parthanatos with apoptosis and combining apoptosis with autophagy and oxidative stress regulation. Occurring concurrently with multiple cell death pathways provides dual-pathway targeting an effective solution for resisting therapeutic resistance, most notably in difficult-to-treat aggressive cancers. Further research needs to find optimal medications that produce synergistic cancer cell death effects with low toxic side effects for advancing advanced multi-target cancer treatments.

## Conclusion and Future Perspective

Both apoptosis and parthanatos establish different yet vital death programs that control cancer development and therapeutic response. Apoptosis functions as a robust caspase-dependent mechanism, but cancer treatments base their approach on this method because numerous tumors manage to avoid apoptosis through elevated anti-apoptotic protein levels or mutated p53 regulatory elements. The cell death mechanism known as parthanatos functions as a caspase-independent pathway through PARP-1 overactivation combined with AIF nuclear movement, representing a new method for destroying resistant cancer cells. Multiple studies indicate that parthanatos functions as a vital mechanism in the development of gliomas, leukemia, and ovarian cancer, and exposure to deoxypodophyllotoxin (DPT) and WIN-55 facilitates parthanatos development in drug-resistant tumors. Several compounds modulating oxidative stress have demonstrated enhanced effects on apoptotic and parthanatic pathways in parallel to the development of the new small molecule ZINC253504760, which shows potential as a treatment for multidrug-resistant (MDR) leukemia cells by inducing parthanatos.

Dual-pathway targeting strategies become new opportunities for cancer treatment because apoptosis interacts with parthanatos. Both tumor cell death and tumor killing reach a higher level when BH3 mimetics are combined with PARP inhibitors because these strategies simultaneously activate apoptosis and parthanatos pathways. Scientists observe that MTH1 inhibitors, when administered together with ROS-inducing agents in cervical cancer, lead to elevated DNA damage with activated parthanatos. The therapeutic value is expanded through natural compounds, including arnicolide D and ursolic acid, because these regulate apoptosis and parthanatos and ferroptosis mechanisms. Thresholds requiring further improvement exist for parthanatos-targeting therapies to deliver targeted therapy without adverse effects in healthy cells. Clinical application of parthanatos remains restricted because no biomarkers are available to determine tumor sensitivity to this mechanism.

Research must focus on discovering the exact molecular factors controlling the apoptosis-parthanatos regulatory switch for better therapeutic intervention. New therapeutic mixtures need evaluation because they should activate parthanatos in treatment-resistant cancer types, and scientists should explore approaches to mitigate mitochondrial impairment and metabolic disturbances to achieve treatment aims. Studies must expand their focus regarding parthanatos applications to explore their effect on neurodegenerative disorders and inflammatory diseases. The use of clinical progress with mechanistic knowledge about apoptosis and parthanatos modulation shows crucial potential to surpass therapy resistance and improve cancer therapy results.

## Declaration

### Competing interests 

The authors declare that they have no competing interests. 

### Funding

This research work was funded by Umm Al Qura University, Saudi Arabia under grant number: 25UQU4310387GSSR03.

### Authors' contributions 

GG, MA, EM and AG: Conceptualization, methodology, software. WHA, KG, MR: Writing - original draft preparation. HA, AR, IK: Data curation, writing - review & editing. SIA: Data curation and methodology. SKS: Methodology, software. All authors read and approved the final manuscript.

## Figures and Tables

**Table 1:**
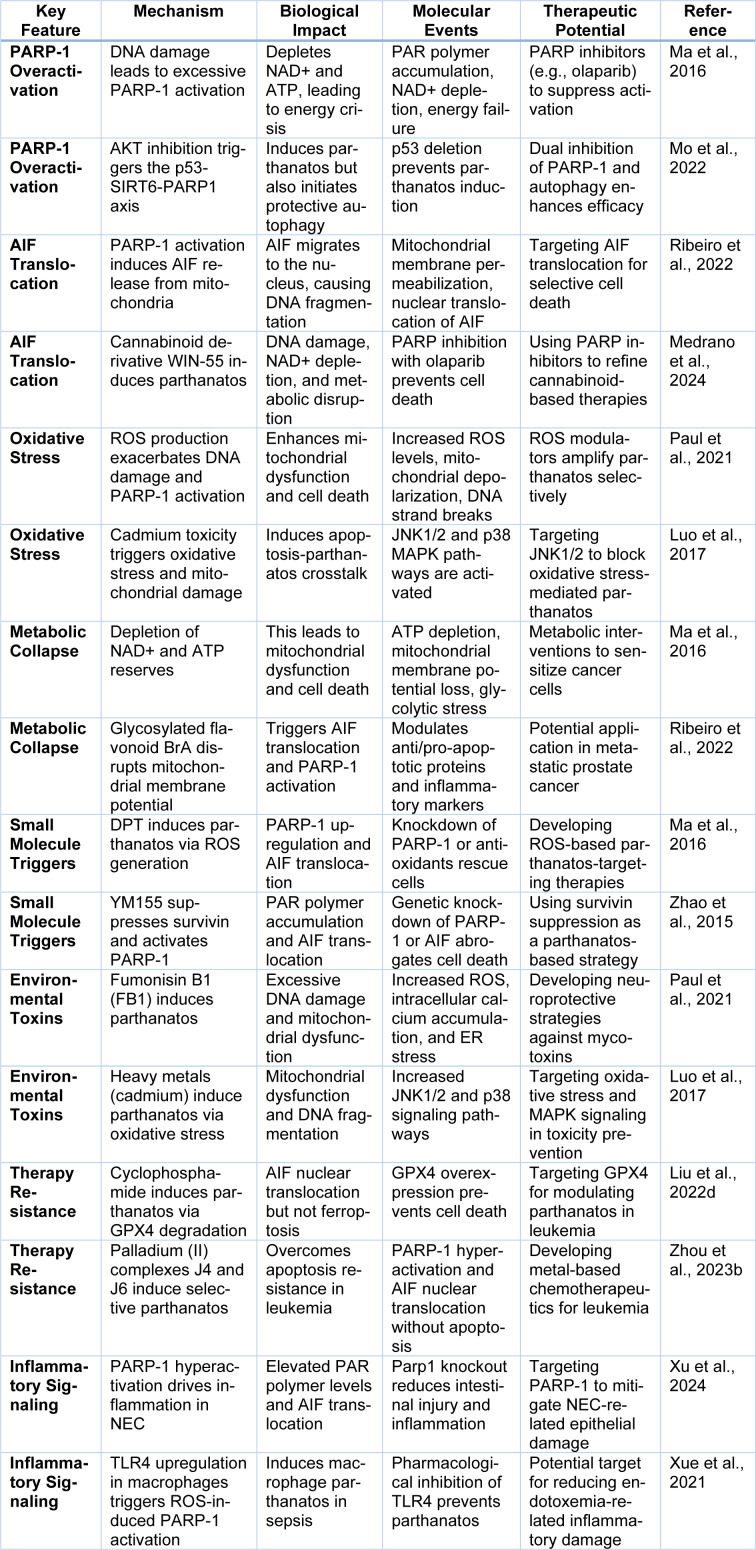
Mechanistic insights of parthanatos, molecular events, and therapeutic strategies

**Table 2:**
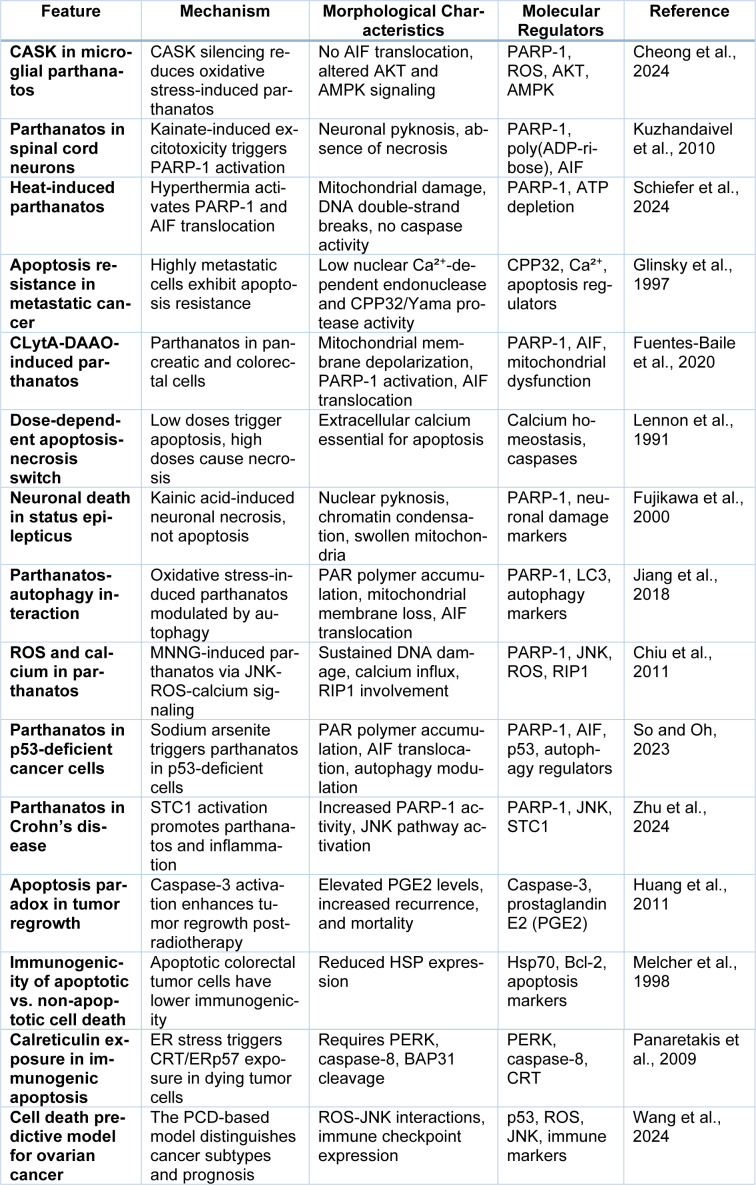
This table compares apoptosis and parthanatos studies, highlighting mechanisms, regulators, and therapeutic implications.
